# Sesquiterpenes from *Laurencia similis*

**DOI:** 10.3390/molecules14051889

**Published:** 2009-05-20

**Authors:** Hua Su, Da-Yong Shi, Jing Li, Shu-Ju Guo, Li-Li Li, Zhao-Hui Yuan, Xiao-Bin Zhu

**Affiliations:** 1 Institute of Oceanology, Chinese Academy of Sciences, Qingdao 266071, China;E-mail: qdshua@sina.com (H.S.); 2 Graduate School of the Chinese Academy of Sciences, Beijing 100049, China; E-mail: yuanzhaohui@ms.qdio.ac.cn (Z-H.Y.), xbzhu@ms.qdio.ac.cn (X-B.Z.)

**Keywords:** *Laurencia similis*, sesquiterpenes, cytotoxic assay

## Abstract

One new sesquiterpene, (4*E*)-1-bromo-5-[(1'S*,3'R*)-3'-bromo-2',2'-dimethyl-6'-methylenecyclohexyl]-3-methylpent-4-ene-2,3-diol (**1**), and fifteen known sesquiterpenes, isopalisol (**2**), luzonensol (**3**), palisadin B (**4**), aplysistatin (**5**), palisadin A (**6**), 4-hydroxyl-palisudin C (**7**), 5-acetoxypalisadin B (**8**), 10-hydroxyaristolan-9-one (**9**), aristol-8-en-1-one (**10**), aristolan-9-en-1-one (**11**), aristolan-1(10)-en-9-one (**12**), aristolan-1(10)-en-9-ol (**13**), aristolan-1(10),8-diene (**14**), aristolan-1,9-diene (**15**) and aristofone (**16**), were isolated from a sample of marine red alga *Laurencia similis*. Their structures were established by detailed NMR spectroscopic analysis and comparison with literature data. Compounds **2**-**9**, and **16** were isolated for the first time from this species. All these metabolites were submitted for a cytotoxicity assay against the tumor cell line BEL7402 (human liver adenocarcinoma), but all of them were found inactive (IC_50_ > 10 μg/mL).

## 1. Introduction

Red algae of the genus *Laurencia* have proved to be a rich source of secondary metabolites, mainly sesquiterpenes, C_15_-acetogenins, and a few di- and triterpenes. More than 300 have been characterized from some 40 species collected in various parts of the world since the 1960s [1]. Many of these metabolites have been reported to possess a variety of biological activities, such as antimicrobial [2], antifeedant [3], anthelmintic [4,5], and cytotoxic activities [6,7].

**Figure 1:**
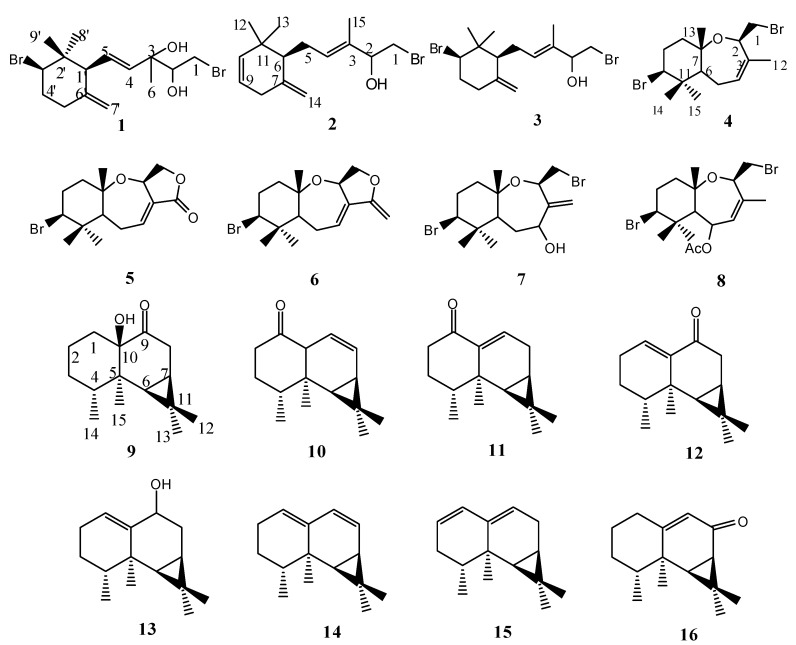
Structure of compounds **1** to **16**.

Within the context of our program to systematically assess the chemical and biological diversity of marine algae distributed along the Chinese coast, further investigations of the chemical constituents of the red alga *L. similis*, collected from Sanya Bay, Hainan Province, led to the isolation and identification of one new sesquiterpene, which is described in this paper, together with 15 known sesquiterpenes.

## 2. Results and Discussion

The dried and powdered alga *L. similis* was extracted with 95% EtOH at room temperature. The concentrated extracts were partitioned between H_2_O and EtOAc. The EtOAc-soluble fraction was purified by a combination of silica gel and Sephadex LH-20 column chromatography, as well as preparative TLC procedures, to yield compounds **1**-**16**.

Compound **1** was isolated as colorless oil, and showed IR absorptions for hydroxy functional group (3410 cm^-1^). The positive ESIMS exhibited a characteristic quasi-molecular ion peak cluster at *m/z* 399/401/403 (1:2:1) [M-H_2_O+Na]^+^, suggested the presence of two bromine atoms in the molecule. The molecular formula was deduced to be C_15_H_24_Br_2_O_2_ from the HRESIMS of the pseudomolecular ion ([M-H_2_O+Na]^+^, 401.1441), which indicated that there were three sites of unsaturation. ^1^H and ^13^C NMR data revealed the presence of two double bonds, both a terminal methylene [*δ*_H_: 4.83 (s, 1H, H-7'α), 4.58 (d, 1H, *J* = 7.2 Hz, H-7'β); *δ*_C_: 146.9 (C-6'), 110.3 (C-7')] and a 3,3-disubstituted propenyl [*δ*_H_: 5.88 (dd, 1H, *J* =9.8, 15.5 Hz, H-5), 5.57(d, 1H, *J* =15.5 Hz, H-4); *δ*_C_: 128.5 (C-5), 136.6 (C-4)], and no any other multiple bonds signals. Thus, it required **1** to be monocyclic. The NMR data (Table 1) demonstrated the presence of three methyls, three methylenes, three methines, and two quaternary carbons in addition to the four olefinic carbons. Comparison of the NMR data of **1** with the Compound **2** suggested that it had the same cyclohexane ring portion. This portion could be connected by HMBC data, including from H_3_-8' and H_3_-9' to C-1', C-2' and C-3', from H_2_-7' to C-1' and C-5'. The third spin system was also established by the HMBC data, including between H_3_-6 and C-2, C-3 and C-4, between H-4 and C-3 and C-1'. Furthermore, the connectivity of C-1' and C-5 was also established by the HMBC data from H-1' to C-4, C-5, C-2', C-6', C-7' and C-8'. Thus, the planar structure of **1** was clearly established.

**Table 1:** ^1^H- and ^13^C-NMR Data of **1**. NMR data were measured at 500 MHz for proton and at 125 MHz for carbon in CDCl_3_ for **1** (*δ* in ppm, *J* in Hz.)**.** Assignments were corroborated by ^1^H, ^1^H-COSY, HMQC, and HMBC experiments.

*Pos.*	1		
	*δ*_H_	*δ*_C_	H→C (HMBC)
1	3.51(t, 11.2), 3.78(dd, 2.1, 11.2)	46.7 (t)	
2	3.69 (dd, *J*=2.1 Hz)	77.6 (d)	
3		74.2 (s)	
4	5.57(d, 15.5)	136.6 (d)	C-3, C-1'
5	5.88 (dd, 9.8, 15.5)	128.5 (d)	
6	1.05 (s)	24.3 (q)	C-2, C-3, C-4
1'	2.52 (d, 9.8)	56.4 (d)	C-4, C-5, C-2',C-6', C-7', C-8'
2'		41.1 (s)	
3'	4.12 (dd, 4.2, 12.0)	66.2 (d)	
4'	2.10 (m), 2.27 (m)	36.4 (t)	
5'	2.10 (m), 2.37 (m)	34.9 (t)	
6'		146.9 (s)	
7'	4.83 (s), 4.58 (d, 7.2)	110.3 (t)	C-1', C-5'
8'	0.93 (s)	16.2 (q)	C-1', C-2', C-3', C-9'
9'	1.34 (s)	29.3 (q)	C-1', C-2', C-3', C-8'

The relative configurations of **1** at C-1' and C-3' were shown to be the same as those of γ-ionone by analysis of a NOESY experiment and detailed NMR data comparison [8,9]. In the 2D NOESY spectrum, the observed correlations of H-1' with H-3' and Me-8' indicated the *cis*-orientation for these protons. The stereochemistry at C-3 and C-4 could not be determined unambiguously. The above spectral evidences established the structure for compound **1**: (4*E*)-1-bromo-5-[(1'S*, 3'R*)-3'-bromo-2', 2'-dimethyl-6'-methylenecyclohexyl]-3-methylpent-4-ene-2, 3-diol.

Compounds **2**-**16** were identified as: isopalisol (**2**), luzonensol (**3**), palisadin B (**4**), aplysistatin (**5**), palisadin A (**6**), 4-hydroxyl-palisudin C (**7**), 5-acetoxypalisadin B (**8**), 10-hydroxyaristolan-9-one (**9**), aristol-8-en-1-one (**10**), aristolan-9-en-1-one (**11**), aristolan-1(10)-en-9-one (**12**), aristolan-1(10)-en-9-ol (**13**), aristolan-1(10),8-diene (**14**), aristolan-1,9-diene (**15**), aristofone (**16**),respectively, by spectroscopic analysis (^1^H-NMR, ^13^C-NMR, and MS) and comparisons with literature data [10,11,12,13,14,15,16,17]. Compounds **2-8** and **16** were isolated from this specimen for the first time.

Compounds **1**–**16** were evaluated for the cytotoxicity against a human liver adenocarcinoma (BEL7402) cell line. However, all of them were found to be inactive (IC_50_ >10 *μ*g/mL).

**Figure 2:**
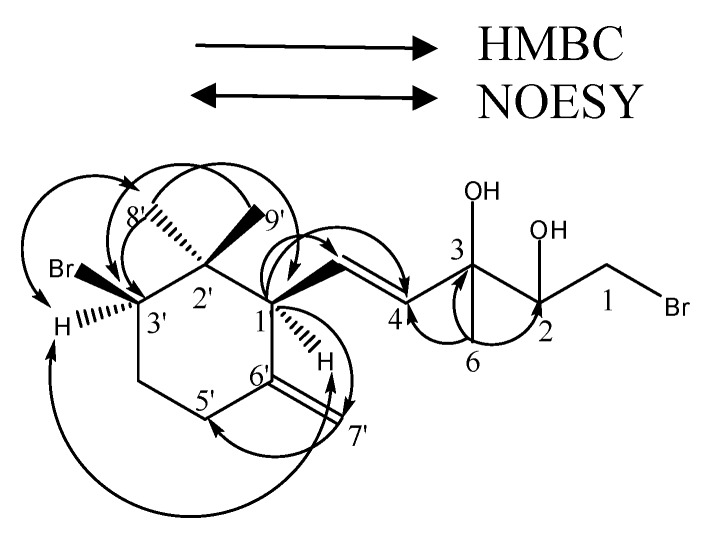
Selected HMBC and NOESY correlations of compound **1**.

## 3. Experimental

### 3.1. General

1D and 2D NMR spectra were obtained on a Bruker Avance500 spectrometer at 500 and 125 MHz for ^1^H and ^13^C, respectively. Mass spectra were collected using a VG Autospec 3000 mass spectrometer. Column chromatography (CC) was performed on silica gel (200–300 mesh, Qingdao Haiyang Chemical Co., Qingdao, China) and Sephadex LH-20 (Sigma). TLC was carried out with precoated silica gel plates (GF-254, Qingdao Haiyang Chemical Co., Qingdao, China).

### 3.2. Alga material

The marine red alga *L. similis* was collected from Hainan coastlines of People’s Republic of China, in May 2006 and identified by Prof. L.-P. Ding at the Institute of Oceanology, Chinese Academy of Sciences (IOCAS). A voucher specimen (No.2006037) was deposited in the Herbarium of Marine Organisms at IOCAS.

### 3.3. Extraction and Isolation

The air-dried alga *L. similis* (1.1 kg) was powdered and extracted with 95% EtOH at room temperature. The resulting extractive solution was filtered and evaporated under reduced pressure (<45 ℃) to yield a dark residue (34.2 g), and then partitioned between H_2_O and EtOAc. The EtOAc phase (28.5 g) was subjected to column chromatography (CC) over silica gel eluting with a gradient of increasing EtOAc in petroleum ether (PE, 0~100%) to give 7 fractions on the basis of TLC analysis. Fraction III (1.9 g) were further chromatographed over Sephadex LH-20 eluting with PE-CHCl_3_-MeOH (5:5:1) to yield compounds **1** (6.8 mg), **2** (5.7 mg), **4** (6.7 mg), **5** (7.3 mg) and **8** (7.3 mg) respectively. Fraction IV (0.6 g) was further chromatographed over Si gel eluting with a gradient of increasing EtOAc (35-100%) in PE to yield **3** (8.3 mg), **6** (7.9 mg), **10** (8.6 mg), **11** (10.0 mg), **14** (5.6 mg) and **16** (5.6 mg). Fraction Ⅴ (3.6 g) was decolored by column chromatography over Sephadex LH-20 eluting with PE-CHCl_3_-MeOH (5:5:1) and preparative TLC (PE/EtOAc 40:3) to afford compound **7** (7.3 mg), **9** (8.3 mg), **12** (11.1 mg), **13** (9.7 mg) and **15** (7.5 mg).

(4E)-1-bromo-5-[(1'S*, 3'R*)-3'-bromo-2', 2'-dimethyl-6'-methylenecyclohexyl]-3-methylpent-4-ene-2, 3-diol (**1**): colorless oil; IR (film) v_max_ 3410, 3012, 2921, 1440, 1065, 990 cm^-1^; [α]20D +18.4 (CHCl_3_, *c* 0.30); ^1^H NMR (CDCl_3_) (see Table 1); ^13^C NMR data (CDCl_3_) (see Table 1). ESI-MS: *m/z* 399/401/403 (1:2:1) [M-H_2_O+Na]^+^. HR-ESI-MS: *m/z* 401.1441([M-H_2_O+Na]^+^) (calcd for 401.1426, C_15_H_22_Br_2_ONa).

*Isopalisol* (**2**): C_15_H_23_BrO, colorless oil; EI-MS *m/z*(%): 282 (3), 280 (3, M-H_2_O), 265 (8), 201 (15), 121 (100), 105 (48), 93 (55); ^1^H-NMR(CDCl_3_, 500MHz) *δ*: 3.45 (2H, m, H_2_-1), 4.22 (1H, t, *J*=5.0Hz, H-2), 5.48 (1H, m, H-4), 2.25 (1H, m, H-5α), 2.90 (1H, m, H-5β), 2.00 (1H, m, H-6), 2.64 (2H, m, H_2_-8), 5.52 (1H, m, H-9), 5.37 (1H, m, H-10), 0.95 (3H, s, H_3_-12), 1.02 (3H, s, H_3_-13), 4.60 (1H, s, H-14α), 4.86 (1H, s, H-14β),1.65 (3H, s, H_3_-15); ^13^C-NMR(CDCl_3_, 125MHz) *δ*: 38.5 (t, C-1), 76.5 (d, C-2), 133.4 (s, C-3), 128.4 (d, C-4), 25.2 (t, C-5), 52.4 (d, C-6), 145.5 (s, C-7), 32.4 (t, C-8), 123.1 (d, C-9), 136.9 (q, C-10), 37.1 (s, C-11), 25.1 (q, C-12), 30.3 (q, C-13), 109.7 (t, C-14), 12.2 (q, C-15).

*Luzonensol* (**3**): C_15_H_24_Br_2_O, colorless oil; EI-MS *m/z*: 364 (3), 362 (6), 360 (3, M-H_2_O), 281 (100), 201 (47),147 (30), 121 (80); ^1^H-NMR(CDCl_3,_ 500MHz) *δ*: 3.49 (1H, dd, *J*=9.0, 10.0 Hz, H-1α), 3.40 (1H, dd, *J*=4.5, 10.0 Hz, H-1β), 4.74 (1H, dd, J=4.5, 9.0 Hz, H-2), 5.29 (1H, m, H-4), 2.45 (1H, m, H-5α), 2.25 (1H, m, H-5β), 1.83 (1H, m, H-6), 2.04 (1H, m, H-8α), 2.35 (1H, m, H-8β), 2.08 (1H, m, H-9α), 2.30 (1H, m, H-9β), 4.15 (1H, dd, J=4.5, 11.5Hz, H-10), 0.87 (3H, s, H_3_-12), 1.20 (3H, s, H_3_-13), 4.56 (1H, s, H-14α), 4.91 (1H, s, H-14β), 1.69 (3H, s, H_3_-15); ^13^C-NMR(CDCl_3_,125MHz) *δ*: 36.9 (t, C-1), 70.1 (d, C-2), 133.1 (s, C-3), 129.9 (d, C-4), 24.7 (t, C-5), 53.2 (d, C-6), 145.3 (s, C-7), 37.2 (t, C-8), 35.6 (t, C-9), 66.7 (d, C-10), 41.6 (s, C-11), 16.6 (q, C-12), 28.4 (q, C-13), 110.0 (t, C-14), 17.4 (q, C-15).

*Palisadin B* (**4**): C_15_H_24_OBr_2_, colorless oil; ESI-MS *m/z*: 378/380/382; ^1^H-NMR (CDCl_3,_ 500MHz) *δ*: 3.41 (1H, dd, *J*=7, 11Hz, H-1α), 3.73 (1H, dd, *J*=3, 11Hz, H-1β), 4.54 (1H, m, H-2), 5.63 (1H, d, *J*=8 Hz, H-4), 2.05 (3H, m, H_2_-5 and H-6), 1.77 (2H, m, H_2_-8), 2.25 (2H, m, H_2_-9), 3.95 (1H, dd, *J*=5, 12 Hz, H-10), 1.27 (3H, s, H_3_-12), 1.36 (3H, s, H_3_-13), 0.95 (3H, s, H_3_-14), 1.15 (3H, s, H_3_-15); ^13^C-NMR(CDCl_3_,125MHz) *δ*: 36.2 (t, C-1), 70.7 (d, C-2), 136.1 (s, C-3), 129.4 (d, C-4), 25.9 (t, C-5), 52.8 (d, C-6), 77.5 (s, C-7), 36.7 (t, C-8), 32.9 (t, C-9), 66.3 (d, C-10), 40.8 (s, C-11), 21.0 (q, C-12), 22.0 (q, C-13), 17.9 (q, C-14), 30.7 (q, C-15).

*Aplysistatin* (**5**): C_15_H_21_BrO_3_, colorless oil; EI-MS *m/z*: 330/328[M]^+^ (1), 300/298 (4), 286/284(8), 249(5), 218/216(8), 204/202(33), 189/187(19), 139(24), 123(100), 107(39); ^1^H-NMR (CDCl_3_, 500MHz) *δ*: 0.96(3H, s, H_3_-13), 1.18(3H, s, H_3_-15), 1.30(3H, s, H_3_-14), 3.86(1H, dd, *J* = 7.0 , 9.0 Hz, H-1α), 3.91(1H, m, H-10), 4.48(1H, t, *J* = 9.0, 9.0 Hz, H-1β), 5.13(1H, m, H-2), 6.95(1H, m, H-4). ^13^C-NMR (CDCl_3_,125MHz) *δ*: 69.9(C-1), 66.8(C-2), 132.0(C-3), 143.0(C-4),27.2(C-5), 51.3(C-6), 79.0(C-7), 32.4(C-8), 37.7(C-9), 65.1(C-10), 41.0(C-11), 169.3(C-12), 18.0(C-13), 30.7(C-14), 21.7(C-15).

*Palisadin A* (**6**): C_15_H_23_BrO_2_, colorless oil; EI-MS *m/z*: 314/316; ^1^H-NMR (CDCl_3,_ 500MHz) *δ*: 1.00(3H, s, H_3_-14), 1.25(3H, s, H_3_-15), 1.37(3H, s, H_3_-13), 1.55(1H, m, H-8α), 1.85(1H, m, H-8β), 2.25(2H, m, H_2_-9), 2.40(2H, m, H_2_-5), 2.40(1H, m, H-6), 3.93(1H, dd, *J* = 8.0, 8.0 Hz, H-1α), 3.95(1H, dd, *J* = 5.0, 12.0 Hz, H-10), 4.10(1H, dd, *J* = 8.0, 8.0 Hz, H-1β), 4.45(2H, m, H_2_-12), 4.83(1H, m, H-2), 5.63(1H, br s, H-4). ^13^C-NMR (CDCl_3_, 125MHz) *δ*: 75.4(C-1), 71.0(C-2), 141.9(C-3), 121.1(C-4), 26.3(C-5), 51.8(C-6), 78.0(C-7), 37.6(C-8), 32.7(C-9), 66.2(C-10), 41.0(C-11), 72.0(C-12), 21.9(C-13), 18.0(C-14), 30.8(C-15).

*4-hydroxyl-Palisudin C* (**7**): C_15_H_24_Br_2_O_2_, colorless oil; EIMS: *m/z* 394 396 398 [M]^+^ (1:2:1), 376 378 380 [M-H_2_O]^+^ (1:2:1); ^1^H-NMR (CDCl_3,_ 500MHz) *δ*: 3.31 (1H, t, *J* = 10.5 Hz, H-1α), 3.44 (1H, dd, *J* = 8.8, 10.5 Hz, H-1β), 4.43 (1H, d, *J* = 7.7 Hz, H-2), 4.86 (1H, t, *J* = 8.1 Hz, H-4), 1.43–1.46 (1H, m, H-5α), 2.20–2.24 (1H, m, H-5β), 1.61–1.62 (1H, m, H-6), 1.59–1.60 (1H, m, H-8α), 1.70–1.73 (1H, m, H-8β), 2.05–2.07 (1H, m, H-9α), 2.22–2.23 (1H, m, H-9β), 3.90 (1H, dd, *J* = 4.3,12.8 Hz, H-10), 4.91 (1H, s, H-12α), 5.23 (1H, s, H-12β), 1.26 (3H, s, H_3_-13), 0.97 (3H, s, H_3_-14), 1.10 (3H, s, H_3_-15); ^13^C-NMR (CDCl_3_, 125MHz) *δ*: 37.3 (t, C-1), 70.9 (d, C-2), 153.2 (s, C-3), 68.8 (d, C-4), 34.9 (t, C-5), 48.8 (d, C-6), 77.5 (s, C-7), 36.9 (t, C-8), 32.3 (t, C-9), 66.1 (d, C-10), 40.3 (s, C-11), 107.1 (t, C-12), 22.3 (q, C-13), 17.8 (q, C-14), 30.8 (q, C-15).

*5-Acetoxypalisadin B* (**8**): C_17_H_26_Br_2_O_3_, colorless oil; EI-MS *m/z*(%): 436/438/440; ^1^H-NMR (CDCl_3_, 500MHz) *δ*: 1.01(3H, s, H_3_-14), 1.20(3H, s, H_3_-15), 1.63(3H, s, H_3_-13), 1.80(3H, s, H_3_-12), 2.08(3H, s, H_3_-OAc), 1.58(1H, m, H-8α), 1.84 (1H, m, H-8β), 1.73(1H, m, H-6), 2.15~2.31(2H, m, H_2_-9), 3.41(1H, dd, *J* = 8.0, 11.0 Hz, H-1α), 3.70(1H, dd, *J* = 3.0, 11.0 Hz, H-1β), 3.87(1H, dd, *J* = 4.0, 12.0 Hz, H-10), 4.44(1H, d, *J* = 10.0 Hz, H-2), 5.69(1H, d, *J* = 8.0 Hz H-4), 5.78(1H, d, *J* = 8.0 Hz, H-5); ^13^C-NMR(CDCl_3_,125MHz) *δ*: [25.2, 170.3(C-OAc)], 34.8(C-1), 70.1(C-2), 142.5(C-3), 127.0(C-4), 69.6(C-5), 53.7(C-6), 77.8(C-7), 39.3(C-8), 32.7(C-9), 66.1(C-10), 41.3(C-11), 21.2(C-12), 21.5(C-13), 18.7(C-14), 30.8(C-15).

*10-Hydroxyaristolan-9-one* (**9**): C_15_H_24_O_2_, colorless oil; FAB-MS(pos.): 236 [M]^+^; ^1^H-NMR (CDCl_3_, 500MHz) *δ*: 0.80(1H, d, *J* = 9.1 Hz, H-6), 0.94(3H, d, *J* = 6.7 Hz, H_3_-14), 1.05(3H, s, H_3_-12), 1.06(3H, s, H_3_-13), 1.20(3H, s, H_3_-15), 1.22~1.33(3H, m, H_2_-3 and H-7), 1.38(1H, m, H-1α), 1.47 (1H, m, H-4), 1.56 (1H, m, H-2α), 1.65 (1H, m, H-2β), 1.92(1H, m, H-1β), 2.26 (1H, d, *J*=17.0 Hz, H-8α), 3.09 (1H, dd, *J* = 9.0, 17.0 Hz, H-8β); ^13^C-NMR (CDCl_3_,125MHz) *δ*: 29.5 (t, C-1), 22.8 (t, C-2), 29.1 (t, C-3), 34.8 (d, C-4), 46.8 (s, C-5), 29.0 (d, C-6),22.9 (d, C-7), 34.5 (t, C-8),215.9 (s, C-9), 77.7(s, C-10), 21.0 (t, C-11), 16.8 (q, C-12), 30.6 (q, C-13),17.0 (q, C-14), 18.4 (q, C-15).

*Aristol-8-en-1-one* (**10**): C_15_H_22_O, colorless oil; FAB-MS (pos.): 218 [M]^+^; ^1^H-NMR (CDCl_3_, 500MHz) *δ*: 0.56 (3H, s, H_3_-15), 0.78 (1H, d, *J* = 8.5 Hz, H-6), 1.01 (3H, s, H_3_-12), 1.05 (3H, d, *J* = 6.6 Hz, H_3_-14), 1.16 (3H, s, H_3_-13), 1.23 (1H, dd, *J* = 5.6, 8.5 Hz, H-7), 1.69 (1H, m, H-3α), 1.94 (1H, m, H-3β), 1.96 (1H, m, H-4), 2.36 (2H, m, H_2_-2), 2.58 (1H, br s, H-10), 5.86 (1H, dd, *J* = 1.9, 10.1 Hz, H-9), 6.02 (1H, m, H-8, H-8). ^13^C-NMR (CDCl_3_, 125MHz) *δ*: 209.9 (s, C-1),40.5 (t, C-2), 31.4 (t, C-3), 40.1 (d, C-4),39.1 (s, C-5), 35.1 (d, C-6), 24.7 (d, C-7), 127.3 (d, C-8), 118.8 (d, C-9), 54.7 (d, C-10), 26.1 (s, C-11), 16.0 (q, C-12),30.5 (q, C-13), 15.5 (q, C-14), 15.9 (q, C-15).

*Aristolan-9-en-1-one* (**11**): C_15_H_22_O, colorless oil; ^1^H-NMR (CDCl_3_, 500MHz) *δ*: 0.71 (1H, d, *J* = 9.4 Hz, H-6), 0.87 (1H, m, H-7), 0.99 (3H, H, s, H_3_-15), 1.03 (3H, s, H_3_-12), 1.06 (3H, d, *J* = 6.8 Hz, H_3_-14), 1.07 (3H, s, H_3_-13), 1.73 (2H, m, H_2_-3), 1.98 (1H, m, H-4), 2.28 (1H, m, H-2α), 2.34 (1H, m, H-8α), 2.50 (1H, m, H-2β), 2.55 (1H, m, H- 8β), 6.41 (1H, m, H-9α). ^13^C-NMR (CDCl_3_, 125MHz) *δ*: 202.0 (s, C-1), 40.0 (t, C-2), 27.8 (t, C-3), 35.9 (d, C-4), 37.8 (s, C-5), 32.0 (d, C-6), 19.3 (d, C-7), 22.5 (t, C-8), 132.5 (d, C-9), 143.5 (s, C-10), 18.8 (s, C-11), 15.3 (q, C-12), 29.8 (q, C-13),15.4 (q, C-14), 22.6 (q, C-15).

*Aristolan-1(10)-en-9-one* (**12**): C_15_H_22_O, colorless oil; ^1^H-NMR (CDCl_3_, 500MHz) *δ*: 0.90 (1H, d, *J* = 9.4 Hz, H-6), 0.96 (1H, m, H-7), 0.99 (3H, s, H_3_-12), 1.02(3H, s, H_3_-15), 1.04 (3H, d, *J* = 5.8 Hz, H_3_-14), 1.05 (3H, s, H_3_-13), 1.50 (2H, m, H_2_-3), 1.76 (1H, m, H-4), 2.24 (2H, m, H_2_-2), 2.66 (1H, dd, *J* = 1.1, 20.0Hz, H-8α), 2.78 (1H, dd, *J* = 6.1, 20.0 Hz, H-8β), 6.71 (1H, m, H-1). ^13^C-NMR (CDCl_3_, 125MHz) *δ*: 136.4 (d, C-1), 25.9 (t, C-2), 26.1 (t, C-3), 36.7 (d, C-4), 37.6 (s, C-5),29.8 (d, C-6), 19.0 (d, C-7), 34.6 (t, C-8), 200.2 (s, C-9), 142.5 (s, C-10),18.7 (s, C-11), 14.8 (q, C-12), 30.1 (q, C-13),15.9 (q, C-14), 24.0 (q, C-15).

*Aristolan-1(10)-en-9-ol* (**13**): C_15_H_24_O, colorless needles ; ^1^H-NMR (CDCl_3_, 500MHz) *δ*: 0.53 (1H, d, *J* = 9.1 Hz, H-6), 0.75 (1H, m, H-7), 0.97 (3H, s, H_3_-12), 1.07(3H, s, H_3_-15), 0.95 (3H, d, *J* = 6.9 Hz, H_3_-14), 0.98 (3H, s, H_3_-13), 1.38 (2H, m, H_2_-3), 1.75 (1H, m, H-4), 2.01 (2H, m, H_2_-2), 1.22 (1H, m, H-8α), 2.32 (1H, m, H-8β), 5.53 (1H, m, H-1). ^13^C-NMR (CDCl_3_, 125MHz) *δ*: 116.4 (d, C-**1**), 25.4 (t, C-**2**), 26.7 (t, C-**3**), 36.9 (d, C-**4**), 38.8 (s, C-**5**), 32.9 (d, C-**6**),18.4 (d, C-**7**), 30.7 (t, C-**8**), 67.4 (d, C-**9**), 145.7 (s, C-**10**), 18.6 (s, C-**11**), 16.1 (q, C-**12**), 29.8 (q, C-**13**),16.6 (q, C-**14)**,23.9 (q, C-**15**).

*Aristolan-1(10)-8-diene* (**14**): C_15_H_22_, colorless needles; ^1^H-NMR (CDCl_3_, 500MHz) *δ*: 0.94 (1H, d, *J* = 8.4 Hz, H-6), 1.17 (1H, dd, *J* = 5.3, 8.3 Hz, H-7), 0.92 (3H, s, H_3_-12), 0.96(3H, s, H_3_-15), 0.99 (3H, d, *J* = 6.9 Hz, H_3_-14), 1.11 (3H, s, H_3_-13), 1.53 (1H, m, H-3α), 1.67 (2H, m, H-3β and H-4), 2.16 (2H, m, H_2_-2), 5.73 (1H, dd, *J* = 5.3, 9.7 Hz,, H-8), 5.28 (1H, m, H-1), 5.85 (1H, d, *J* = 9.7Hz, , H-9). ^13^C-NMR (CDCl_3_, 125MHz) *δ*: 122.0 (d, C-**1**), 25.2 (t, C-**2**), 27.7 (t, C-**3**), 35.8(d, C-**4**), 34.6 (s, C-**5**), 34.5 (d, C-**6**), 24.4 (d, C-**7**), 124.3 (d, C-**8**), 126.7 (d, C-**9**), 141.7 (s, C-**10**), 26.3(s, C-**11**), 14.7(q, C-**12**), 29.3 (q, C-**13**), 15.9 (q, C-**14)**, 23.0 (q, C-**15**).

*Aristolan-1,9-diene* (**15**): C_15_H_22_, colorless needles; ^1^H-NMR (CDCl_3_, 500MHz) *δ*: 0.67 (1H, d, *J* = 9.3 Hz, H-6), 0.81 (1H, dd, *J* = 7.1, 9.3 Hz, H-7), 1.03 (3H, m, H_3_-12), 0.96(3H, s, H_3_-15), 0.99 (3H, d, *J* = 6.9 Hz, H_3_-14), 1.04 (3H, m, H_3_-13), 1.90 (1H, dd, *J* = 11.2, 18.3 Hz, H-3α), 2.01(1H, m, H-3β), 5.55 (1H, dd, *J* = 5.6,9.8Hz, H-2), 2.23(1H, dd, *J* = 5.3, 20.1 Hz,, H-8α), 2.55(1H, dd, *J* = 7.1, 20.1 Hz, H-8β), 5.88 (1H, d, *J* = 9.8 Hz, H-1), 5.26 (1H, m, H-9). ^13^C-NMR (CDCl_3_, 125MHz) *δ*: 129.1(d, C-1), 125.2 (d, C-2), 32.3 (t, C-3), 34.1(d, C-4), 35.1 (s, C-5), 31.4 (d, C-6),19.7 (d, C-7), 22.4 (t, C-8), 122.7 (d, C-9),140.7 (s, C-10), 18.3(s, C-11),15.0(q, C-12), 29.9 (q, C-13),15.1 (q, C-14), 21.1 (q, C-15).

*Aristofone* (**16**): C_15_H_22_O_,_ colorless needles; ^1^H-NMR (CDCl_3_, 500MHz) *δ*: 1.29~1.36 (3H, m, H_2_-1 and H-3β), 1.48 (1H, m, H-2β), 1.74~.178 (2H, m, H-2α and H-3α), 1.65 (1H, d, *J* = 8.0Hz, H-4), 2.18 (1H, d, *J* = 8.0 Hz, H-6), 2.34~2.40 (1H, m, H-7), 5.64 (1H, s, H-9), 1.40 (3H, s, H_3_-12), 1.92 (3H, s, H_3_-13), 0.99 (3H, dd, *J* = 2.4, 6.8 Hz, H_3_-14), 1.12 (3H, s, H_3_-15). ^13^C-NMR (CDCl_3_,125MHz) *δ*: 33.0 (t, C-l), 30.4 (t, C-2), 26.0 (t, C-3), 38.5 (d, C-4), 39.4 (s, C-5), 39.0(d, C-6), 35.4 (d, C-7), 195.9(s, C-8), 124.1 (d, C-9), 167.3 (s, C-l0), 24.1 (s, C-11), 16.2 (q, C-12), 29.5 (q, C-13), 22.4 (q, C-14), 16.3 (q, C-15); EIMS: 218 [M]^+^.

### 3.4. Cytotoxicity Assay

Cytotoxic assay toward the human liver adenocarcinoma (BEL7402) cell line was carried out as previously reported [18].
